# Prefrontal Internal Event‐Driven Analysis of Dynamical Electroencephalographic Biomarkers in Depression During Emotional Auditory Task

**DOI:** 10.1111/cns.70382

**Published:** 2025-04-16

**Authors:** Qinglin Zhao, Kunbo Cui, Hua Jiang, Zhongqing Wu, Lixin Zhang, Mingqi Zhao, Bin Hu

**Affiliations:** ^1^ Gansu Provincial Key Laboratory of Wearable Computing, School of Information Science and Engineering Lanzhou University Lanzhou People's Republic of China; ^2^ School of Medical Technology Beijing Institute of Technology Beijing People's Republic of China; ^3^ CAS Center for Excellence in Brain Science and Intelligence Technology Shanghai Institutes for Biological Sciences, Chinese Academy of Sciences Shanghai People's Republic of China; ^4^ Joint Research Center for Cognitive Neurosensor Technology of Lanzhou University & Institute of Semiconductors, Chinese Academy of Sciences Lanzhou People's Republic of China

**Keywords:** depression, EEG, emotional auditory task, event‐related analysis, prefrontal cortex

## Abstract

**Aims:**

This study for the first time proposed a novel prefrontal internal event‐driven analytic framework for electroencepalography (EEG) data, which aim to dynamically resolve neural processes during natural emotional auditory tasks.

**Methods:**

The framework employed a novel unsupervised time‐series clustering model for internal prefrontal event extraction, which supports event‐related analyses with the absence of external event labeling. The framework was validated using a 64‐channel EEG data obtained from 110 (55 depressed) subjects in a three‐polar (positive, neutral, and negative) emotional‐auditory task.

**Results:**

Our results suggest that anhedonia in depressed patients are associated with high activation levels in multiple brain regions during specific internal events, and we found that cross‐frequency modulation of the bilateral prefrontal lobe with other relevant regions revealed completely different unidirectional patterns for the positive and negative tasks.

**Conclusion:**

Our study confirmed the effectiveness of the framework in resolving fine‐grained internal event‐driven neural processes without relying on traditional precise event‐related data acquisision paradigms that often require high attention on the task events and causes high cognitive load. Our study present new insights for identifying dynamical electroencephalographic biomarkers in depression, which potentially provide EEG signal decoding solutions for EEG feedback‐based closed‐loop intervention of depression.

## Graphical abstract

We first proposed a novel framework for prefrontal internal event‐driven EEG data analysis, which permits event‐related analysis for experimental paradigms with low cognitive load and absence of external task events, and reveals internal event‐related neural oscillatory power specific to depressed emotional processes as well as abnormal low‐frequency phase‐to‐high‐frequency amplitude modulation.

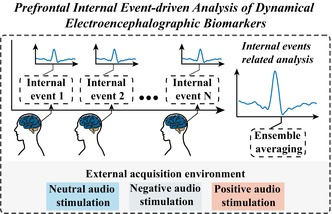

## 1 Introduction

Depression is a mental disorder that causes great worldwide disease burden [1], which affects many aspects of life, such as social relationships at school and at work. In particular, depression is one of the main reasons for suicide, while suicide leads to more than 700,000 deaths each year [2]. Studies on the diagnosis and treatment of depression have increasingly recognized mood dysfunction as an essential factor in depression [3], including negative mood preference and deficits in emotion regulation [4, 5]. In this aspect, the prefrontal cortex (PFC), due to its key role in emotional functioning [6], has been considered one of the key brain regions for the study of affective dysfunction in depression [7, 8]. However, in electroencephalography (EEG)‐based studies, the insufficiency of more dynamic and emotional state‐specific analytical tools often limits finer interpretations of prefrontal signals and their interactions with other brain regions associated with dysfunctional emotion processing of depressed patients.

Currently, the imbalances between cortical excitation and inhibition in the PFC of depressed patients potentially mediate key pathological mechanisms, [9, 10] which can potentially benefit both diagnostic and therapeutic applications. In diagnostic studies, a number of studies confirmed the effectiveness of prefrontal EEG signals in classifying depressed patients from the healthy. For example, Cai et al. [11] constructed 270 linear and nonlinear feature matrices from prefrontal three‐channel Fp1, Fp2, Fpz EEGs and achieved 79.27% accuracy in depression classification using a K‐nearest neighbor classifier. Shen et al. proposed an improved empirical modal decomposition algorithm to extract prefrontal EEG features, which achieved more than 81% depression recognition accuracy on all four independent EEG data sets [12]. Tian et al. [13] achieved 90.7% depression recognition accuracy by developing a prefrontal three‐lead EEG sensor combined with Ant Lion Optimization and KNN classifiers. On the other hand, in many noninvasive neuromodulation‐based therapeutic studies, subregions in the PFC have been frequently targeted for brain stimulation, of which the relatively low response/remission rates (response rates of 41%–56% and remission rates of 26%–28%) lead to limited clinical application [14, 15, 16]. One potential reason for such a situation could be that the effect of neuromodulation is not only related to the pattern of stimulation but also depends on the functional states of the brain during the stimulation [17]. In other words, dynamic neuromodulation with a high temporally specific emotional processing state can potentially result in enhanced therapeutic effects.

**FIGURE 1:**
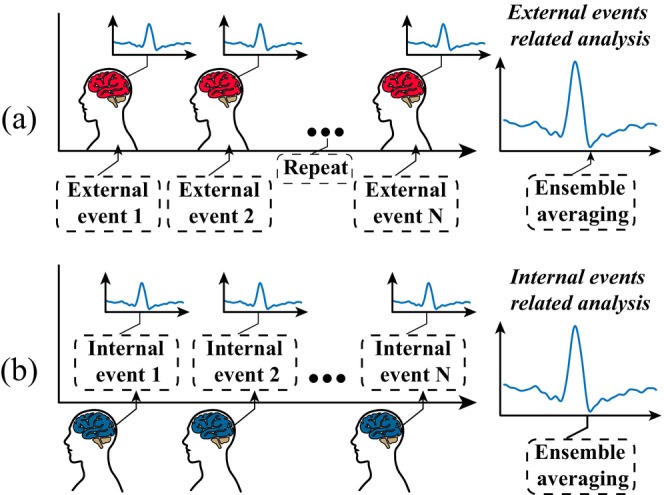
Illustration of event‐related analysis. (a) External event‐related analysis paradigm. (b) Internal event‐related analysis paradigm.

In laboratory environments, emotional processes are often induced with various auditory, visual, or lingual tasks. In experimental paradigms with precise temporal events, tasks are often designed to be strictly attached to certain external events (see Figure 1a), which permit analyses of neural dynamics in response to these tasks. For instance, event‐related power change of neural oscillations, also known as event‐related desynchronization/synchronization (ERD/ERS), reveals changes in recruitment or excitability of local neural networks related to a certain event [18]. For example, Segrave et al. [19] explored patterns of frequency band activity during working memory processing in patients with major depression and found that depressed patients performing effortful cognitive processing required additional neural resources to reach normal levels. Another study based on the affective Go/No‐go paradigm noted that improvement in depressive symptoms with positive cognitive therapy was associated with changes in desynchronized activation in the alpha frequency band [20]. Besides, connectivity metrics such as cross‐frequency coupling (CFC) during an event reveal changes in information exchanges associated with the event [21]. For example, Riddle et al. [22] used a reward paradigm to study the prefrontal cortex in depressed patients and found that depression modulates prefrontal top‐down control patterns in different symptom dimensions. Although these analyses can resolve emotional process‐associated neural dynamics with relatively high temporal precision, their experimental designs are less natural and typically require particular focus from the participants on the external task events [19, 23]. Such high cognitive loads prevent actual application of these paradigms to depressed patients, especially in the cases of clinical diagnosis and therapy [24, 25]. Accordingly, one persuasive need is the development of an approach for effectively resolving emotional neural dynamics from more naturalistic task paradigms without precise external events (see Figure 1b). However, there remain three main obstacles to such application: (1) the lack of a computational framework for internal‐event‐related analyses; (2) the lack of a method for the extraction of interpretable internal events in the absence of external events; and (3) the lack of validity of such an approach in the study of prefrontal dysfunction in depression.

To address the above‐mentioned issues, our study for the first time proposed a novel prefrontal internal cortical event‐driven analysis framework for the dynamical analysis of neural processes in depression during emotional auditory tasks. Specifically, we defined prefrontal internal events according to localized few‐channel (Fp1, Fpz, and Fp2 according to 10–20 system) prefrontal microstates. The localized prefrontal microstates were identified using a Gaussian mixture generative model within a variational Bayesian framework in a data‐driven manner [26, 27]. To verify our approach, we recruited 55 depressed and 55 healthy participants and acquired 64‐channel EEG data from these participants with their eyes closed under a three‐polar (positive, negative, and neutral) emotional auditory task [11, 28]. The auditory task paradigm was designed without precise events in order to maintain low cognitive load for both the depressed and the healthy participants. With the internal events extracted according to the prefrontal signals using our approach, we examined event‐related neural dynamics in terms of power change in neural oscillations and cross‐oscillation interaction in a reconstructed brain source space. Overall, our study presents new insights for the dynamical brain functional state‐specific electroencephalographic biomarkers in depression in a data‐driven approach. In addition, our study may also provide potential signal decoding solutions for EEG feedback‐based closed loop neuromodulation therapy of depression.

The main contributions of our study are summarized as follows:

1. For the first time, we proposed a novel framework for prefrontal internal event‐driven EEG data analysis, which permits event‐related analysis for experimental paradigms with low cognitive load and absence of external task event.
2. We proposed a prefrontal internal event extraction algorithm, which automatically identifies internal event markers from localized few‐channel EEG data.
3. We demonstrated the validity of our framework on a large‐scale depression data set in resolving the abnormal neural dynamics associated with the PFC in depressed patients and in revealing internal event‐related neural oscillatory power specific to depressed emotional processes, as well as abnormal low‐frequency phase to high‐frequency amplitude modulation.

## 2 Materials and Methods

**FIGURE 2:**
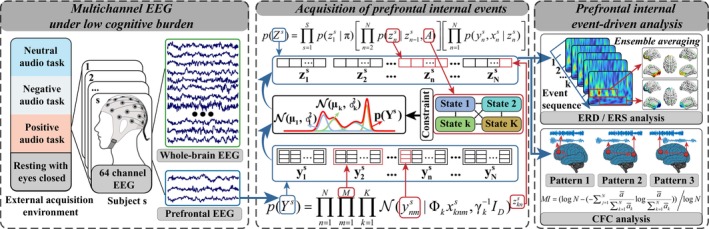
Illustration of prefrontal internal event‐related analysis. Prefrontal few‐channel EEG was first obtained from a multichannel EEG data set. Subsequently, discrete states were extracted from prefrontal few‐channel EEG by an unsupervised temporal clustering model and then prefrontal internal event sequences were obtained. Finally, internal prefrontal events were used as markers for event‐related desynchronization/synchronization (ERD/ERS) analysis and cross‐frequency coupling (CFC) analysis, respectively.

The proposed prefrontal internal event‐driven framework (see Figure 2) is summarized as follows: (1) acquiring prefrontal EEG from multichannel EEG in a low cognitive load paradigm, (2) constructing a temporal unsupervised clustering model to extract prefrontal internal events, and (3) applying prefrontal internal events to event‐related desynchronization/synchronization (ERD/ERS) analysis and cross‐frequency coupling (CFC) analysis. We will present the corresponding steps in the following subsections.

### 2.1 Experiment and Data

We recruited 55 subjects with depressive disorder (the DD group, age 35.41 ± 10.78 years; 23 females) and 55 healthy control subjects (the HC group, age 35.78 ± 11.06 years; 26 females) to participate in this study. Subjects were screened by specialized physicians according to the International Neuropsychiatric Interview (MINI) and the Diagnostic and Statistical Manual of Mental Disorders (DSM‐IV; 70 cases in the Second People's Hospital of Gansu Province and 40 cases in the Second Xiangya Hospital of Central South University). Participants in the depression group were not undergoing any drug therapy or psychological therapy. Detailed information about the participants and the inclusion/exclusion criteria can be found in Appendix S1. The DD group had significantly higher PHQ‐9 [29] scores than the HC group (DD = 17.16 ± 5.41, HC = 1.23 ± 1.38, *t* = 21.143, *p* < 0.001).

During the experiment, a subject, who sat relaxed and comfortably on a chair, was asked to keep awake with their eyes closed and avoid eye and body movements. The subject performed a three‐polar (neutral, negative, and positive) emotional auditory task. The task consisted of 12 experimental trials, 4 for each emotional polarity. Each trial contained a 6‐s soundtrack, followed by a 6‐s interval for rest. The sound tracks were selected from the IADS‐2 database [28], of which the details (e.g., audio name, playback order, scoring details) are provided in Table 1. During the experiment, we recorded a total of 144 s EEG data for each subject using a 64‐channel EEG amplifier (Acti CHamp, BrainProducts, Germany) at a 1 kHz sampling rate, with the physical reference set to the FCz electrode. The whole experimental procedures were approved by the Ethical Committee of the Second Xiangya Hospital, Central South University (Project No. 2015BA113B02, approval date: May 18, 2016). The experimental procedures were agreed to by each participant with a written informed consent before the experiment and were conducted in accordance with the 1964 Helsinki declaration and its later amendments.

**TABLE 1:** Experimental paradigm for audio stimulation.

Playback sequence	Audio name	Properties	Valence		Arousal		Activate	
			Mean	Variance	Mean	Variance	Mean	Variance
1	Clock	Neutral	4.34	1.42	3.51	1.42	4.64	2.06
2	Alarm	Neutral	4.30	2.50	6.99	2.50	3.87	2.02
3	Buzzer	Negative	2.42	1.62	7.98	1.62	2.84	2.11
4	Alarm Clock	Negative	2.78	1.93	7.54	1.93	3.95	2.24
5	Slot Machine2	Positive	7.32	1.64	6.56	1.64	6.39	2.30
6	Robin	Positive	7.12	1.56	4.47	1.56	5.73	1.92
7	Rattle Snake	Neutral	3.55	1.99	6.98	1.99	3.50	1.82
8	Battle Taps	Neutral	3.02	2.06	5.34	2.06	3.67	1.99
9	Puppy	Negative	2.88	2.14	6.40	2.14	3.80	2.17
10	Child Abuse	Negative	1.57	1.43	7.27	1.43	3.49	2.48
11	Seagull	Positive	6.95	1.64	4.38	1.64	5.91	1.80
12	Rain1	Positive	5.84	1.73	3.93	1.73	5.70	1.89

The EEG data were high‐pass filtered at 1 Hz and low‐pass filtered at 80 Hz using zero‐phase finite impulse response (FIR) filters and downsampled to 250 Hz. The data were then corrected for bad channels by interpolating channels from their neighboring channels. Subsequently, the Independent Component Analysis algorithm was applied to manually inspect and remove remaining ocular and myogenic artifacts [30]. In the last step, the data were average re‐referenced for further analysis.

### 2.2 Internal Event Extraction

#### 2.2.1 Unsupervised Time‐Series Clustering Model

Previous studies have demonstrated the effectiveness of identifying depressed patients according to prefrontal scalp EEG (e.g., recorded from the Fp1, Fpz, and Fp2 electrodes) [12, 31]. To more dynamically evaluate the prefrontal neural dynamics in depression, our study used a data‐driven approach to extract prefrontal internal events from signals recorded at the Fp1, Fpz, and Fp2 electrodes. Specifically, we employed a Gaussian mixture model within a variational Bayesian framework to obtain prefrontal localized microstates. An internal event was identified at the moment when a microstate transferred to a different one.

In this Gaussian mixture model, we assume that the prefrontal EEG data are a collection of time‐series changes in the modeled observed variables, and that the prior probability distribution of the modeled observed variables is represented by a two‐layer sparse probability distribution [32]. In this case, the data within the same observation window of length M are assumed to be generated by the same prefrontal microstate, and the changes in the data in neighboring windows are constrained by the first‐order Markov transfer matrix [33, 34]. Let $y_{n}^{s}=\left(y_{n1}^{s},y_{\mathrm{nm}}^{s},\ldots ,y_{\mathrm{nM}}^{s}\right),n=1,2,\ldots ,N,s=1,2,\ldots ,S$ denotes the EEG data of subject s in the *n*th time window of length M, where $y_{\mathrm{nm}}^{s}$ is a D‐dimensional vector, and D denotes the number of EEG electrodes. Each $y_{n}^{s}$ correspond to a state variable $z_{n}^{s}$, which is a K‐dimensional binary vector ($\sum_{K}z_{\mathrm{nk}}^{s}=1,K$ is the number of hidden states). Accordingly, the marginal distribution of $y_{n}^{s}$ is described as Equation (1):

(1) $$p\left(y_{n}^{s}|x_{n}^{s},z_{n}^{s}\right)=\prod_{m=1}^{M}\prod_{k=1}^{K}N{\left(y_{\mathrm{nm}}^{s}|\Phi_{k}x_{\mathrm{knm}}^{s},\gamma_{k}^{-1}I_{D}\right)}^{z_{\mathrm{kn}}^{s}},$$

where Φ is the linear transformation matrix. $x_{n}^{s}$ is assumed to obey a Gaussian distribution with zero mean and variance ω, $x_{n}^{s}\sim \prod_{m=1}^{M}N\left(0,{\left(\mathrm{diag}\left(\omega_{m}\right)\right)}^{-1}\right)$. The noise follows the Gaussian distribution $e_{n}\sim N\left(0,\gamma^{-1}I_{D}\right)$ with zero mean and variance of γ. Here $I_{D}$ denotes the D‐dimensional unit matrix and $\mathrm{diag}\left(∙\right)$ denotes the transformation of the vector into a diagonal matrix.

For a given initial state π and transfer matrix A, the conditional probability distribution of the prefrontal microstate variable Z is expressed as Equation (2):

(2) $$\begin{array}{c} p\left(Z\right)=\prod_{s=1}^{S}p\left(z_{1}^{s}|\pi \right)\left[\prod_{n=2}^{N}p\left(z_{n}^{s}|z_{n-1}^{s},A\right)\right]\left[\prod_{n=1}^{N}p\left(y_{n}^{s},x_{n}^{s}|z_{n}^{s}\right)\right]. \end{array}$$

Variational inference was used to optimize the posterior probability distribution of the parameters [26, 27], and a forward‐backward inference algorithm to optimize the prefrontal microstate variable Z [35] as well as a Viterbi algorithm for generating the optimal path to state Z [36]. Details on the derivation of the model prior probability distribution are given in Appendix S1.

Note that the optimal window length M of the observed data and the optimal number of prefrontal microstates K should be defined according to data. For the selection of the window length *M*, we determined the correlation between the sampling points with an interval of *M* by calculating the correlation (Pearson correlation) separately. For the number of state categories *K*, it is determined by the Bayesian Information Criterion (BIC) [37], the calculation process is shown in Equation (3), where ${\mathrm{Num}}_{\Omega }$ is the number of model parameters. The $\Omega =\left[\gamma ,\Phi ,v,\omega ,\pi ,A\right]$ denotes the list of model parameters.

(3) $$\begin{array}{c} \begin{array}{c} \mathrm{BIC}=-2\ln\left(p\left(Y|X,Z\right)\right)+{\mathrm{Num}}_{\Omega }\ln\left(S*N\right). \end{array} \end{array}$$

#### 2.2.2 Model Inference and Training

In variational inference [26], given the observation data Y and prior distribution, our goal is to minimize the Kullback–Leibler (KL) divergence between the variational posterior distribution Q and the true posterior distribution $p\left(X,Z,\Omega |Y\right)$ as in Equation (4).

(4) $$\begin{array}{c} Q^{*}≜\min_{Q}\mathrm{KL}\left[Q\|p\left(X,Z,\Omega |Y\right)\right]. \end{array}$$

The log‐likelihood of the observed data Y is expressed in variational inference as Equation (5).

(5) $$\begin{array}{c} \ln p\left(Y\right)=L\left(Q\right)+\mathrm{KL}\left[Q\|p\left(X,Z,\Omega |Y\right)\right]. \end{array}$$

Here $L\left(Q\right)$ denotes the variational lower bound function, it is shown that minimizing $\mathrm{KL}\left[Q\|p\left(X,Z,\Omega |Y\right)\right]$ is equal to maximizing the variational lower bound $L\left(Q\right)$. Applying the mean field theory, the variational posterior distribution Q is expressed in the form of a product of posterior distributions (see Equation (7)).

(6) $$L\left(Q\right)=E\left[\ln\frac{p\left(Y,X,Z,\Omega \right)}{Q}\right],$$

(7) $$\begin{array}{c} \begin{array}{c} Q=q\left(X|Z\right)q\left(Z\right)\prod_{i=1}^{G}q_{i}\left(\Omega_{i}\right). \end{array} \end{array}$$

As a result, the optimal log‐posterior distribution of the parameter $\Omega_{i}$ is shown in Equation (8). The $E_{/Q_{i}}\left[∙\right]$ denotes the expectation for parameters outside of the parameter $Q_{i}$, and Const indicates a constant.

(8) $$\begin{array}{c} \ln p\left(Q_{i}^{*}\right)=E_{/Q_{i}}\left[\ln p\left(Y,X,Z,\Omega \right)\right]+\text{Const} \end{array}.$$

Subsequently, we compute the parameters of the posterior distribution for each $Q_{i}$ in turn according to Equation (8).

The labels *Z* can be initialized by the K‐means algorithm, which further determines the initial assignments of π and A. The expected value $z_{n}^{s}$ for each sample in different hidden states k is assigned as the inverse normalized value of the distance from each sample to the cluster centers. The hyperparameters of the Dirichlet distribution and the Gamma distribution are initially assigned as $10^{-4}$, whereas the remaining parameters can be randomly initialized.

The variational lower bound $L\left(Q\right)$, which increases during the training iterations, determines whether the algorithm converges. The training process converges when the increment of $L\left(Q\right)$ approaches zero. The expectation of the log‐likelihood distribution of the lower bound $L\left(Q\right)$ is expressed in Equation (9).

(9) $$\begin{array}{c} L\left(Q\right)=E\left[\ln p\left(Y,X,Z,\Omega \right)\right]-E\left[\ln p\left(X,Z,\Omega \right)\right] \end{array}$$

#### 2.2.3 Internal Event Definition

Finally, the internal event sequence corresponding to the EEG data sequence Y is determined by the state sequence Z computed by the unsupervised time‐series clustering model. Specifically, the set of internal events is the set of basic elements of the state sequence Z (the number of elements is K), and a single internal event segment Ie is defined as a duration segment with the same state Z. For example, the internal event sequence of subject s is shown in Equation (10), where $z_{\mathrm{lh}}^{s}$ denotes the hth element of the lth microstate fragment.

(10) $$\begin{array}{c} \begin{array}{c} \text{Event}\_{\text{sequence}}^{s}=\left[{\mathrm{Ie}}_{1}^{s},{\mathrm{Ie}}_{2}^{s},{\mathrm{Ie}}_{3}^{s},\ldots ,{\mathrm{Ie}}_{l}^{s}\right],l=1,2,\ldots ,L<N,\\ \text{where}\;{\mathrm{Ie}}_{l}^{s}=\left[z_{l1}^{s},z_{l2}^{s},\ldots ,z_{\mathrm{lh}}^{s}\right],h=1,2,\ldots ,H<N. \end{array} \end{array}$$

### 2.3 Source Signal Reconstruction

To minimize the effect of volume conduction, the analysis after event extraction was performed in the brain source space. The source signals were reconstructed using the Noninvasive Electrophysiology Toolbox [38]. Specifically, the 64‐channel EEG data, along with a template head structural magnetic resonance (MR) image in the MNI space and the electrode positions, were fed into the toolbox. In this toolbox, the template head model was created by first co‐registering the electrode to the head surface, then segmenting the MR image into 12 tissue layers (skin, eyes, muscle, fat, spongy bone, compact bone, cortical/subcortical gray matter, cerebellar gray matter, cortical/subcortical white matter, cerebellar white matter, cerebrospinal fluid, and brain stem) with the MR‐TIM software [39], and finally calculating the lead field matrix using the SimBio method with source dipoles positioned in the 6‐mm hexahedral meshes. The template head model and the individual 64‐channel EEG data were fed into the exact low‐resolution brain electromagnetic tomography (eLORETA) algorithm [40], resulting in reconstructed three‐directional source signals for 4940 gray matter voxels. For each voxel, a single‐dimensional signal was obtained according to the first principal component of the three directions [41, 42].

### 2.4 Internal Event‐Related Analysis

#### 2.4.1 Characteristics of the Internal Events

We evaluated overall properties of the neural signal according to the prefrontal internal events in terms of absolute amplitude, total field power (TFP) [43], correlation between Fp1 and Fp2 (Pearson correlation coefficient), phase lock value (PLV) [44] between Fp1 and Fp2, duration of the microstates, and coverage of the microstates, where TFP and PLV are calculated from Equations (11) and (12).

(11) $$\mathrm{TFP}\left(t\right)=\sqrt{\frac{1}{D}\sum_{d=1}^{D}{\left(y_{d}\left(t\right)-\text{mean}\left(y\left(t\right)\right)\right)}^{2}}.$$

(12) $$\begin{array}{c} \mathrm{PLV}=\left|\frac{1}{N*M}\sum_{t=1}^{N*M}e^{\Phi_{\mathrm{Fp}1}\left(t\right)-\Phi_{\mathrm{Fp}2}\left(t\right)}\right|. \end{array}$$

#### 2.4.2 Power of Neural Oscillations

We assessed the power change of neural oscillations associated with these prefrontal events regardless of participant groups and emotional tasks for the most relevant frequency bands as mentioned previously [45, 46]: theta band (4–7 Hz), alpha band (8–12 Hz), and beta band (13–30 Hz). Specifically, the neural signal of each voxel was filtered between these frequency bands using zero‐phase filters (order 500, Blankman window). The mean power of each band‐limited signal within each event trial (from the start of the event to the end of the event) were assessed by subtracting its corresponding baseline that was defined as the mean power of that band across the whole task range. The mean powers of each type of events were averaged and resulted in brain maps for each neural oscillation and events. Let $O_{b}^{s}$ be the EEG power signal in the frequency band b by subject s. The event‐related synchronization/desynchronization of internal event k on frequency band b is calculated as Equation (14), where R is the power baseline, $O_{\mathrm{lh},b}^{s}$ is the power signal on frequency band b corresponding to the hth element within the lth event segment, and $L_{k}$ is the length of the event sequence corresponding to internal event k.

(13) $$R_{b}^{s}=\frac{1}{N*M}\sum_{t=0}^{N*M}O_{t,b}^{s},$$

(14) $$\begin{array}{c} {\mathrm{Avg}}_{k,b}^{s}=\frac{1}{L_{k}}\sum_{l=0}^{L_{k}}\left(\sum_{h=0}^{H}\left(O_{\mathrm{lh},b}^{s}-R_{b}^{s}\right)\right). \end{array}$$

To further examine the emotional task‐specific differences of such power change between the DD group and the HC group, we repeated the above‐mentioned process for each auditory emotional task. To compare the difference between the two groups, we first generated a set of healthy baseline maps by averaging the brain maps across subjects in the HC group for each frequency band, emotional task and events. We then subtracted the corresponding healthy baseline maps from each individual map in the DD group. The statistical significances were tested using the one‐sample *t*‐test, corrected for multiple comparison using the false discovery rate (FDR) method at the significance level of p<0.001.

#### 2.4.3 Cross‐Frequency Couplings

In addition to power change, we also evaluated cross‐frequency couplings associated with these prefrontal events in 46 region of interests (ROIs). Specifically, we divided the brain into regions according to the standard automated anatomical labeling (AAL) template [47], and formed 2 bilateral PFC ROIs that were, respectively, consisted of corresponding unilateral dorsolateral superior frontal gyrus, middle frontal gyrus, and medial superior frontal gyrus. We also selected 44 additional ROIs outside the PFC (see Table S1) from these AAL regions, which are either related emotional processes or related to auditory tasks. For each ROI, a single neural signal that represent its major neural dynamics was obtained according to the first principal component of all the signals within this ROI [41, 42]. The neural signal of each ROI was band filtered to theta, alpha, and beta oscillations, in order to test modulation effects of low‐frequency phase (i.e., theta) on the high‐frequency amplitude (i.e., alpha and beta) using the phase–amplitude modulation index (MI) [48]. We individually evaluated the modulation indices of the two PFC ROIs to examine the interactions within each of these regions. We also evaluated the modulation indices of the bilateral PFC signals to the 44 additional ROIs, and of these additional ROIs to the bilateral PFC signals. Similar to the previous section, we subtracted the cross‐subject average modulation indices of the HC group from each individual MI of the DD group for each emotional task condition and frequency pair (i.e., theta–alpha and theta–beta), and tested the significant difference between the two groups using an one‐sample *t*‐test, corrected for multiple comparison with the FDR method at the significance level of p<0.001. The MI is calculated as in Equation (15), J is the total number of bands (in this study, *J* took the value of 18), $\bar{a}$ is the average amplitude of individual bands, and $H\left(∙\right)$ denotes the Shannon entropy [48].

(15) $$\begin{array}{c} p\left(j\right)=\frac{\bar{a}}{\sum_{i=1}^{N}\bar{a_{i}}},\\ H\left(p\right)=-\sum_{j=1}^{N}p\left(j\right)\log p\left(j\right),\\ \mathrm{MI}=\frac{\log J-H\left(p\right)}{\log J}. \end{array}$$

#### 2.4.4 Statistical Methods

We employed the Shapiro–Wilk test to assess the normality of the data. For data that followed a normal distribution, we conducted parametric tests including *t*‐tests and analysis of variance (ANOVA), while for data that did not meet the normality assumption, we applied Fisher‐Z transformation to make the data distribution closer to a normal distribution. For the data that eventually did not pass the normality test, we used the Mann–Whitney *U* test as a nonparametric alternative. Additionally, in the cases of multiple comparisons, we applied the FDR correction method.

## 3 Result

### 3.1 Model Hyperparameter Selection

**FIGURE 3:**
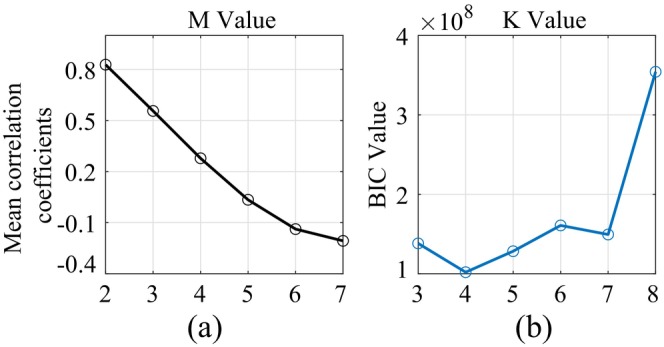
Model hyperparameter selection. (a) Average correlation coefficients with *M* ranging from 2 to 7; (b) BIC (Bayesian Information Criterion) values with *K* ranging from 3 to 8.

**FIGURE 4:**
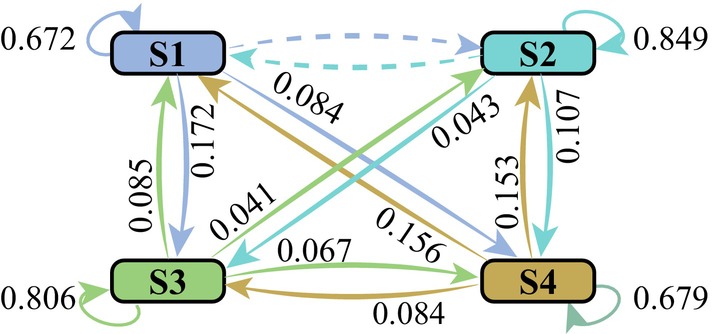
Transfer probabilities across different states. S1, S2, S3, and S4: Internal events identified by the Gaussian generative microstate model.

The hyperparameter M of the model was determined by the correlation between the data sampling points, as shown in Figure 3a the correlation coefficients with *M* ranging from 2 to 7. We chose M=2 for our model, where the correlation coefficient was acceptably high (0.83). The hyperparameter *K* of the model was determined by the BIC of the corresponding model, as shown in Figure 3b the BIC values with *K* ranging from 3 to 8. We chose K=4 for our model where the BIC had the lowest value. According to the model with above parameters, we identified four prefrontal microstates, defined as S1 to S4, of which the transfer matrix of the Markov process was obtained by model training (see Figure 4). The mean transfer probabilities across different states ($p_{\text{interstate}}=0.0993$) were much smaller than the mean transfer probability of a state itself ($p_{\text{instate}}=0.7518$), and no state transfer was seen between states S1 and S2.

### 3.2 Properties of Prefrontal Events

**FIGURE 5:**
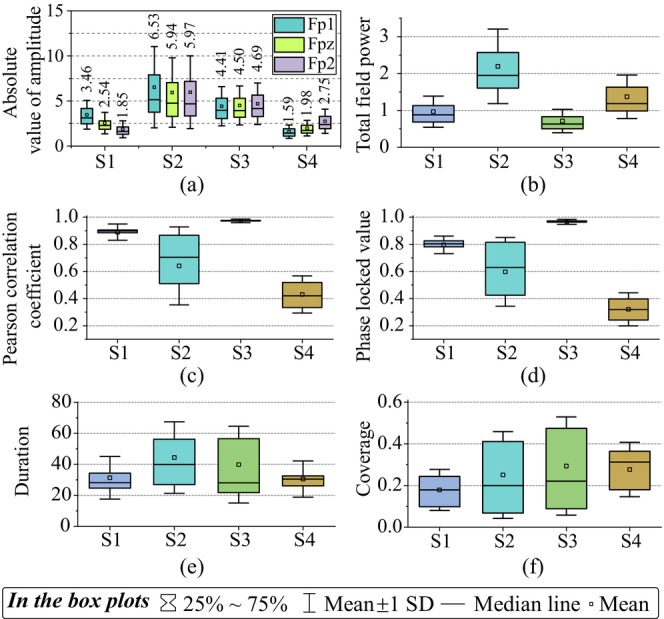
Prefrontal event properties at the sensor level. (a) Mean absolute intensity of each channel and event; (b) mean overall field intensity of each event; (c) mean Pearson correlation between Fp1 and Fp2 for each event; (d) mean phase locked value between Fp1 and Fp2 for each event; (e) average duration of each event; (f) coverage of each event. S1, S2, S3, and S4: Internal events identified by the Gaussian generative microstate model.

The prefrontal events, as calibrated by the microstate generation model, generally showed differences in basic attributes (see Figure 5). Specifically, for the mean intensities (see Figure 5a, $S1_{\text{mean}}=2.618$, $S2_{\text{mean}}=6.144$, $S3_{\text{mean}}=4.535$, $S4_{\text{mean}}=2.106$), we found significant difference across events (p<0.001, two‐way ANOVA), but no significant difference across prefrontal channels (p=0.286, two‐way ANOVA). We also found significant interactions between events and prefrontal channels (p<0.001, two‐way ANOVA). In addition, we found significant difference in the total field power (see Figure 5), the bilateral correlation (see Figure 5c), the bilateral phase locked value (see Figure 5d), the duration (see Figure 5e), and the coverage (see Figure 5f) across events (p<0.001 for each attribute, tested with one‐way ANOVAs). The mean duration of each event (see Figure 5e) was 39.65 ms, with S3 having the longest duration ($S3_{\text{duration}}=50.01$ ms) while S4 having the shortest duration ($S4_{\text{duration}}=31.41$ ms). For the coverage of each state (see Figure 3f), the S3 state had the highest coverage ($S3_{\text{coverage}}=0.293$), and S1 had the lowest coverage ($S1_{\text{coverage}}=0.178$).

### 3.3 Prefrontal Event‐Related Neural Oscillations

**FIGURE 6:**
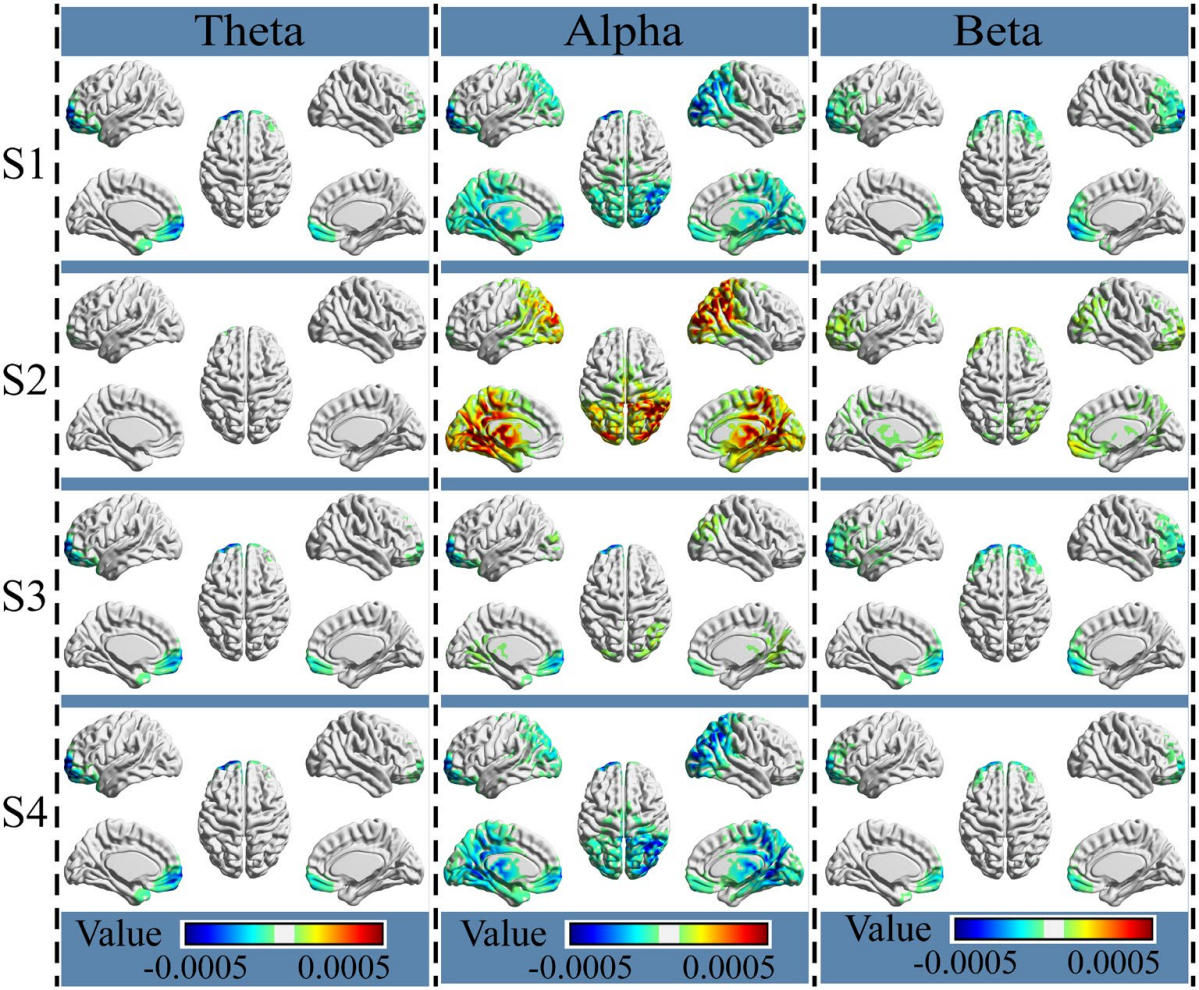
Prefrontal event‐related neural power change of each event. Participant group and emotional auditory tasks were neglected in the analysis. The brain maps show power changes in each band against the corresponding baselines. Red‐ and yellow‐colored regions indicate power increase against the baseline, whereas blue‐ and green‐colored regions indicate power decrease against the baseline. S1, S2, S3, and S4: Internal events identified by the Gaussian generative microstate model.

The analysis of neural oscillations regardless of participant group generally showed a specific distribution of neural power changes associated with each prefrontal event against the overall baselines (see Figure 6). Specifically, for the events S1 and S4, we observed a strong power decrease bilaterally covering the OFC and PFC in all three oscillations, as well as an additional power decrease bilaterally covering large areas around the parietal lobe, occipital lobe, and thalamus in beta oscillations. For the event S2, we observed a strong power increase in the alpha oscillation bilaterally located around large areas of the parietal lobe, occipital lobe, and thalamus, as well as a light beta power increase covering the bilateral OFC, bilateral PFC, and a small part of the right occipital lobe, but no clear theta power change. For the event S3, we observed a strong power decrease at the bilateral OFC and PFC in the theta and beta bands, while there was a slight power increase at the right parietal lobe.

**FIGURE 7:**
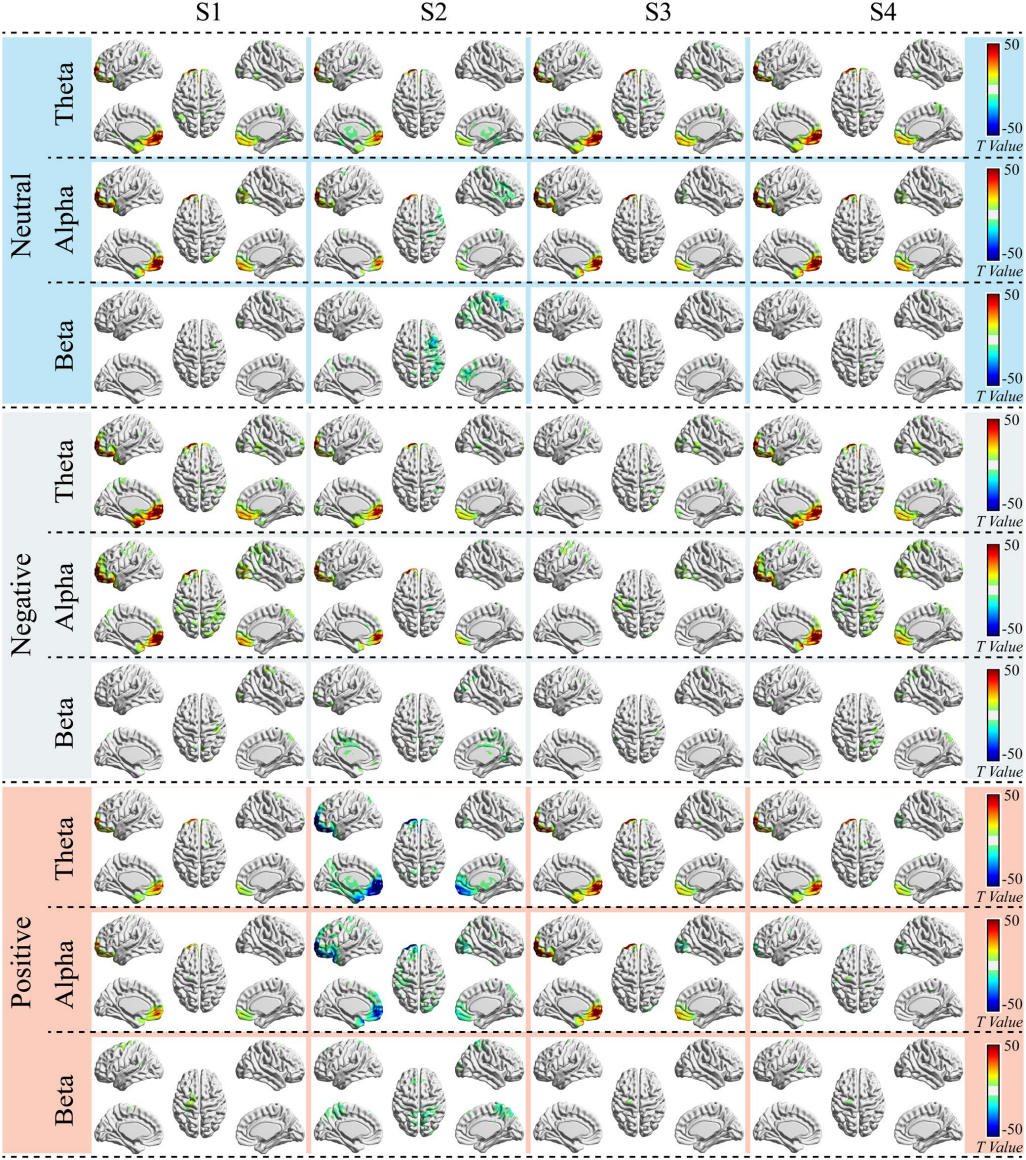
Emotional auditory task‐specific differences in prefrontal event‐related neural power change between the DD group and the HC group. The brain maps are statistical *t*‐maps thresholded at p<0.001, corrected for false discovery rate (FDR). Red‐ and yellow‐colored regions indicate significant higher power in the DD group, whereas blue‐ and green‐colored regions indicate significant lower power in the DD group. DD, depressive disorder; HC, ealthy control; S1, S2, S3, and S4, internal events identified by the Gaussian generative microstate model.

We further examined differences in such prefrontal event‐related power changes between the DD group and the HC group for each emotional auditory task (see Figure 7). Specifically, for the S1 events, we observed higher power in the DD group during positive emotional tasks at the medial OFC and the ventral medial PFC in theta and alpha oscillations. Similar differences were stronger and extended to the superior and inferior OFC during the neutral emotional tasks and were even more intensified and expanded to the temporal poles during the negative emotional tasks. For the S2 events, we mainly observed strong lower power in the DD group during positive emotional tasks at the OFC, the ventral medial PFC, the rostrolateral PFC, the thalamus, and the temporal poles in theta and alpha oscillations. Such differences were reversed in the DD group during the negative and neutral emotional tasks at similar but reduced brain regions. For the S3 events, we observed higher power in the DD group at the medial OFC, the ventral medial PFC, the superior and inferior OFC, and the temporal poles during positive and neutral emotional tasks in the theta and alpha oscillations. Similar spatial patterns also appeared for the S4 events during neutral and negative emotional tasks in theta and alpha oscillations, as well as during positive emotional tasks in theta oscillation. In general, regions with significant differences bilaterally corresponded to the OFC, the PFC, the temporal pole, thalamus, some parts of the superior parietal cortex, and the anterior cingulate cortex (ACC). It should also be noted that differences in these regions mostly presented bilateral asymmetrical spatial patterns.

### 3.4 Prefrontal Event‐Related Cross‐Frequency Couplings

**FIGURE 8:**
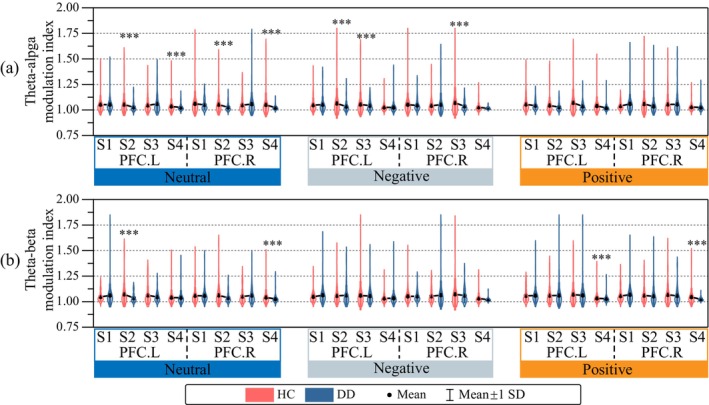
Emotional task‐specific phase–amplitude modulation effects within prefrontal cortex (PFC) region of interests. (a) Theta–alpha modulation indices for left PFC and right PFC; (b) theta–beta modulation indices for left PFC and right PFC. DD, epressive disorder; HC, ealthy control; S1, S2, S3, and S4, internal events identified by the Gaussian generative microstate model; “***” comparisons with significant difference (*p* < 0.001).

The modulation effects of the low‐frequency phases to high‐frequency amplitudes within PFC generally revealed significant lower modulation indices in the DD group than the HC group (see Figure 8). Specifically, in the neutral emotional tasks, significant lower theta–alpha modulations were found for the S2 and S4 events both at the left PFC and the right PFC (Figure 8a Neutral). Such differences were also observed for theta–beta modulations during S2 at the left PFC and during S4 at the right PFC (Figure 8b Neutral). In the negative emotional tasks, significant lower theta–alpha modulations were found for the S2 and S3 events at the left PFC and for the S3 events at the right PFC (Figure 8a Negative). Such differences in negative tasks were not observed for theta–beta modulations (Figure 8b Negative). In the positive emotional tasks, significant lower modulations were only observed for theta–beta modulations during S4 at the left PFC and the right PFC (Figure 8b Negative). Significant lower modulation effects of low‐frequency phase to high‐frequency amplitude were also observed in the DD group between pairs of the PFC ROIs and other related ROIs (see Figure 8).

**FIGURE 9:**
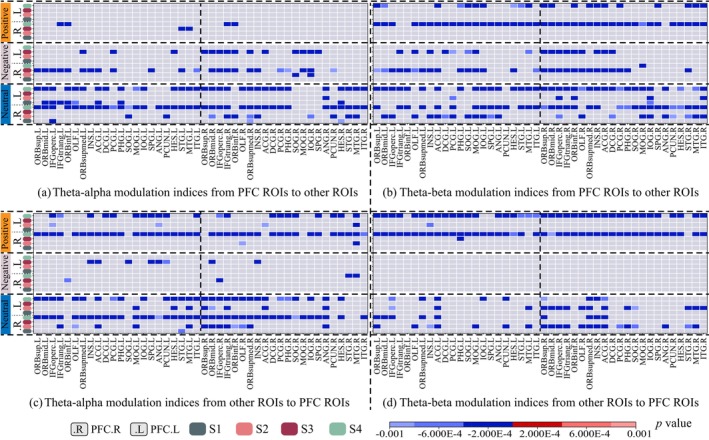
Emotional task‐specific phase–amplitude modulation effects of region of interest (ROI) pairs between prefrontal cortex (PFC) other regions. (a) Theta–alpha modulation indices from PFC ROIs to other ROIs. (b) Theta–beta modulation indices from PFC ROIs to other ROIs. (c) Theta–alpha modulation indices from other ROIs to PFC ROIs. (d) Theta–beta modulation indices from other ROIs to PFC ROIs. Red regions indicate significant higher modulation indices in the DD group, whereas blue regions indicate lower modulation indices in the DD group. The significances were tested with one‐sample *t*‐test at p<0.001, corrected for multiple comparison with the false discovery rate (FDR) method.

In general, significantly reduced modulations most frequently appeared for the S4 events during positive (Figure 9b–d positive) and neutral (Figure 9a–d neutral) emotional tasks, except for the theta–alpha modulations from PFC ROIs to other ROIs during the positive emotional task where the value reduced only for very few OFC regions (Figure 9a positive). Significantly reduced modulation also frequently presented for the S3 events during negative (Figure 9a–c negative) emotional tasks, except for the theta–beta modulations from other ROIs to PFC ROIs (Figure 9d negative). Occasionally, reduced modulations also appeared for the S2 events during neutral tasks. In addition, the reduction of phase–amplitude modulations showed unidirectional patterns. For example, during positive tasks, reductions appeared at fewer regions for the PFC–other direction than for the other–PFC direction in theta–alpha modulations (Figure 9a,c positive, 6 regions vs. 66 regions); conversely, during the negative tasks, reductions appeared at more regions for the PFC–other direction than for the other–PFC direction in theta–alpha (Figure 9a,c negative 39 regions vs. 12 regions) and theta–beta (Figure 9b,d negative, 46 regions vs. 0 regions) modulations. In the remaining cases, such reductions were more or less bidirectional.

## 4 Discussion

### 4.1 Effectiveness of Our Emotional Auditory Tasks

Our study employed auditory emotional tasks with three emotional polarities: neutral, negative, and positive, which brought several benefits as compared to emotional paradigms in other modalities. First, different from the affective visual paradigms [49], our task design allows for closed‐eye acquisition [11], which avoids the presence of excessive ocular disturbances during prefrontal scalp EEG acquisition. Second, instead of traditional event‐related paradigms such as event‐related potential that typically require precise events and responses [50], our auditory tasks worked in a less‐restricted approach. This permitted minimizing potential cognitive load over the depressed subjects, as they might find it difficult to complete complex tasks due to their symptoms [51]. Third, our tasks contained three‐polar emotional stimuli from the International Auditory Stimulus Standards database [28], which supported the probe of emotional process‐specific neural dynamics associated with depression as compared to various resting state‐based studies [52]. In addition to these benefits, our previous studies have demonstrated the effectiveness of such a paradigm. For example, EEG feature‐based classification studies were able to effectively discriminate between depressed and normal subjects [11, 12, 13].

### 4.2 Internal Events From Prefrontal Signals

We investigated whole‐brain neural activity associated with changes in prefrontal EEG signals, as the prefrontal cortex has been reported to be closely associated with emotional processing [6] and is one of the key brain regions in the study of depression‐induced emotional dysfunction [7, 8]. Our study quantified changes in prefrontal neural dynamics as a sequence of internal events according to localized prefrontal electrodes (i.e., Fp1, Fpz, and Fp2), which followed a similar idea to traditional microstate analysis [53, 54]. The microstate analysis typically reflects spontaneous shifts of resting‐state EEG from one brain network activation to another [55]. Our study focused on the whole‐brain activation and network patterns associated with a more localized prefrontal region, similar to a previous study where microstates of the VTA‐Striatal‐Prefrontal Loop were investigated [54]. Our study considered the localized states as internal events, in which the concept of internal events is common in sleep EEG analysis [56]. Such internal events permitted event‐related analyses in the absence of external event markers, in order to reveal richer and more reliable information about dynamics in cortical activations [57] and in large‐scale communications [21].

Our study identified the prefrontal events according to localized prefrontal EEG channels, which required methods that recognize such quasi‐steady‐state structural sequences with relatively few channels. In this respect, we used a Gaussian mixture model within a variational Bayesian framework to discretize the EEG signals into a time‐ordered combination of a finite set of events [27], whose switching is constrained by a first‐order Markov transfer matrix [34], and influenced by the EEG of the sampling point at the moment corresponding to the event [26]. Our experimental results on the three‐channel prefrontal signals demonstrated the existence of four internal prefrontal events and suggested that switches of these events were regular over time, rather than random mutations. We further assessed the properties of these events according to the energy and the cross‐frequency coupling and observed asymmetrical patterns for the events: power in events S1 and S2; relatively low correlation and low phase locked value between Fp1 and Fp2 in event S4. This is consistent with previous findings of asymmetries between left and right brain activity in the prefrontal cortex [58], except that our results suggest that the asymmetry in neural activity in the left and right prefrontal cortex varies with temporal patterns. We should additionally note that our novel methodology of using internal event‐related analysis should be universal, rather than only for depression studies. The extraction of such internal events should not be confined only to the localized prefrontal EEG channels but can be research question‐specific and across the whole brain. For example, for depression studies, we can also focus on the events of precuneus and cingulate gyrus [59]; for motor functional studies, we can focus on the events of motor function‐related cortices.

### 4.3 Emotional Auditory Task‐Specific Dynamical Patterns in Depression

One of the critical obstacles in analyzing neural dynamics is their temporal and spatial coupling in functional brain networks [60]. In our study, events extracted from prefrontal neural signals temporally discretized whole‐brain EEG data, permitting the dissociation of the spatial patterns of brain networks for each emotional auditory task and for each internal event. This is in contrast to previous studies where they directly analyzed EEG recordings of a subject as a whole [11, 12]. Our study dynamically evaluated two types of patterns: prefrontal event‐related neural oscillations and prefrontal event‐related cross‐frequency couplings, in which the former assessed the changes in cortical excitement similar to ERD/ERS, while the latter evaluated interactions between brain regions. These analyses generally revealed emotional auditory task‐specific abnormalities related to these internal events in depression.

The changes of power in neural oscillations, as revealed by previous ERD/ERS studies, reflect cortical activations related to a certain type of events. In detail, a decreased power of a neural oscillation within a local neural network suggests an increased level of cortical recruitment or involvement in a task, as such decrease may be caused by more random firings of local neurons; contrarily, power increases suggest a reduced level of activations [18, 61]. The superimposition of the band‐limited brain maps identified internal event‐specific involvement of different brain regions: S1 and S4 events showed activation in regions covering the OFC and PFC mediated by all three oscillations as well as activation around the parietal lobe, occipital lobe, and thalamus mediated by beta oscillation. The S2 event presented deactivation of the parietal lobe, occipital lobe, and thalamus mediated by alpha oscillation as well as deactivation of the bilateral OFC, bilateral PFC, and a small part of the right occipital lobe mediated by beta oscillation. The S3 event only showed activation in the bilateral OFC and PFC mediated by theta and beta oscillation as well as slight deactivation in the right parietal lobe mediated by the same oscillation. On this basis, our study also revealed rich information about emotional task‐ and internal event‐specific abnormalities in depressed patients compared to healthy participants. For example, in the S2 event during positive emotional tasks, depressed patients showed significantly higher levels of activation mediated by theta oscillations in brain regions corresponding to the OFC, the ventral medial PFC, the rostrolateral PFC, the thalamus, and the temporal poles; conversely, in the S3 events during the same emotional tasks, depressed patients showed significantly lower levels of activation mediated by the same oscillation at the OFC, the ventral medial PFC, the rostrolateral PFC, and the temporal poles but not the thalamus. Interestingly, our study can reveal effects of emotional polarity over the level of activations. For instance, in S2 events during neutral and negative tasks, depressed patients showed significantly decreased levels of activation mediated by the three oscillations, whereas in the same event during positive tasks, depressed patients showed significantly increased levels of activation in the same oscillations. This enhanced activation during positive stimuli may be explained by the previously reported pleasure deficit in depressed subjects [62, 63]. Notably, the regions with significant differences also showed bilateral asymmetrical spatial patterns, which are generally consistent with previous findings that neural interactions in the prefrontal cortex are lateralized in depressed patients [64, 65] and predominantly in the left prefrontal cortex [66]. Overall, the identified brain regions are largely in accordance with results from neuroimaging studies [67, 68], but dynamically reveal more detailed emotional process‐specific and brain state‐specific spatial and spectral patterns.

The changes in cross‐frequency coupling, as revealed by the modulation effects of low‐frequency amplitudes on high‐frequency amplitudes, reflect interactions between brain regions related to a certain type of events. Cross‐frequency cross‐coupling is thought to be the neural basis for the coordination of different brain networks [21], in which the coupling in theta oscillation has been demonstrated to reflect therapeutic effects in transcranial magnetic stimulation [69]. Our study revealed a significant reduction in cross‐frequency interactions within the PFC for depressed patients, which were not bilaterally symmetrical in specific emotional tasks and events. For example, during negative tasks, the event S2 showed a significant reduction in the modulation index of theta phase over alpha amplitude in the left PFC, whereas it showed a nonsignificant conversed pattern in the right PFC. Indeed, such asymmetrical prefrontal neural activity in depression has been frequently reported by previous studies [70, 71]. Interestingly, the cross‐frequency modulations of the bilateral PFC with other related regions revealed completely different unidirectional patterns between positive tasks and negative tasks. For the theta–alpha modulation, positive tasks presented less reduced interactions for the PFC–other direction; conversely, the negative tasks presented more reduced interactions for the same direction. For the theta–beta modulation, the negative task showed more reduced interactions for the PFC–other direction than the opposite direction. Reasonable inference could be that such unidirectional effects may mediate the negative mood preference of depressed patients [14, 72].

As our findings suggest more dynamical emotional prefrontal event‐related, task‐specific, internal, and neural oscillation‐dependent abnormalities in the depressed brain, it may be interesting to further link such differences with pathological findings at the molecular level, particularly the imbalanced neurotransmitter systems. Indeed, changes in brain activation and connectivity in depressed patients could be related to altered levels of the major excitatory and inhibitory systems in the brain, primarily disrupted glutamate‐excitatory and GABA‐inhibitory systems [73]. For example, studies observed reduced glutamate transmission in PFC subregions of depressed patients [9, 74], probably due to altered dendrite length and branching of hippocampus CA3 pyramidal neurons and mPFC layers II/II and V pyramidal neurons when exposed to acute or chronic stresses [75, 76, 77]. Evidences also revealed decreased levels of the GABA synthetic enzyme GAD67 in the PFC [78, 79], as well as reductions of GABA markers in depressed patients [80]. Nevertheless, further studies with more sophisticated experimental designs and multi‐modality data acquisition are still required to draw clearer conclusions on such prefrontal event‐related, task‐specific, internal, and neural oscillation‐dependent brain activity and connectivity alterations. Moreover, it is also crucial to expand our current methodological developments and neuroscientific findings to diagnostic and therapeutic studies for more individualized and targeted diagnosis and neuromodulations. In this respect, feature engineering, systematic model designing, and validating studies should be conducted to ensure the identification of biomarkers with high sensitivity and specificity; more powerful online/real‐time event detection models can be trained and validated with deep learning neural networks [81]; more detailed therapeutic experimental paradigms remain required.

### 4.4 Limitations

There are some limitations in our current study. First, our current method for prefrontal internal event extraction was based on broadband prefrontal signals; further development of methods that take neural oscillations may result in more reliable internal event detection. Second, our current study extracted internal events only from the prefrontal cortex, while existing studies suggest the use of other essential depression‐related brain regions, such as the precuneus and cingulate gyrus [14]. Third, our current internal event extraction employed broadband EEG signal; an in‐depth study of neural oscillation‐dependent internal event extraction approaches may permit finer and more interpretable captions of the internal status of emotional task‐related neural responses. Fourth, our current study focused on the investigation of depression‐related abnormalities driven by internal events but did not assess the potential possibility of such findings improving neuromodulation‐based therapy. Fifth, further extensive feature engineering, as well as model designing and validating studies, remains required to extract and test the sensitivity and specificity of potential biomarkers. Finally, our current results were obtained from a relatively small group of participants. Although the sample size is comparable to many previous studies and generated a sufficient statistical power, further recruitment of more participants with different depression severities and subtypes can ensure not only more reliable and generalizable findings but also potential differentiation of severities and subtypes.

## 5 Conclusion

In this study, we explored activation and interactions across brain regions during three‐polar auditory emotional tasks in healthy and depressed participants, which were dynamically driven by internal prefrontal events extracted from prefrontal EEG signals using an unsupervised Gaussian mixture model. Our results suggest that such prefrontal events can be used to resolve abnormal neural dynamics in depression and revealed emotional process‐specific and internal event‐related abnormalities in the power of neural oscillations and in modulations of low‐frequency phases to high‐frequency amplitudes. Overall, our study demonstrates an effective approach for dynamic analyses of EEG data acquired during tasks without precise external events and may further contribute to the decoding of EEG signals in targeted and personalized closed loop neuromodulation therapies for depression.

## Author Contributions

Qinglin Zhao and Kunbo Cui were responsible for the organization and coordination of the trial. Kunbo Cui and Mingqi Zhao were the chief investigators and responsible for the data analysis. Qinglin Zhao, Kunbo Cui, and Mingqi Zhao developed the trial design. Kunbo Cui, Hua Jiang, Zhongqing Wu, and Lixin Zhang were responsible for visualizing the data and validating the results. Qinglin Zhao, Mingqi Zhao, and Bin Hu acquired funding for the project. All authors contributed to the writing of the final manuscript and contributed to the management or administration of the trial.

## Conflicts of Interest

The authors declare no conflicts of interest.

## Supporting information


Appendix S1.

## Data Availability

The data that support the findings of this study are available from the corresponding author upon reasonable request.
