# Supplementary material for: Prefrontal Internal Event‐Driven Analysis of Dynamical Electroencephalographic Biomarkers in Depression During Emotional Auditory Task
Source: CNS Neurosci Ther. 2025 Apr 16;31(4):e70382. doi: 10.1111/cns.70382 (PMC12001428; doi:10.1111/cns.70382)
Supplement: Supplementary file 1 — Appendix S1. [file CNS-31-e70382-s001.pdf]

Supplementary material

Prefrontal Internal Event-driven Analysis of Dynamical Electroencephalographic Biomarkers in Depression during Emotional Auditory Task

A. Subject inclusion and exclusion criteria

Subjects were screened by specialized physicians according to the International Neuropsychiatric Interview (MINI) and the Diagnostic and Statistical Manual of Mental Disorder. Detailed inclusion and exclusion criteria for the depressed and control groups are shown in Fig. S1.

	Depressed subjects	Normal subjects
Inclusion Criteria	1. Inpatient or outpatient, between the ages of 18 and 55, male or female.<br>2. Elementary school or above literacy level.<br>3. Using the MINI cardboard to meet the diagnostic criteria for depression in the United States Diagnostic Manual of Disease Classification, Fourth Edition (DSM-IV), with a score of>5 on the PHQ-9.<br>4. Not taking any psychotropic medication for 2 weeks prior to enrollment.<br>5. The patients themselves or their legal guardians signed an informed consent form.	1. Be between the ages of 18 and 55, male or female.<br>2. No previous or current established diagnosis of mental disorder.<br>2. Have an elementary school education or above.<br>4. Normal intellectual activity.<br>5. Signed informed consent.
Exclusion criteria	1. Previous or current psychotic disorders.<br>2. Depressive disorders due to organic mental disorders.<br>3. Persons with serious and unstable physical illnesses who are unable to complete the questionnaire and examination and assessment.<br>4. Those with a history of alcohol or psychoactive substance abuse or dependence within one year.<br>5. Pregnant and breastfeeding women who are currently taking birth control pills.<br>6. Those with serious suicidal tendencies.<br>7. Other diseases with organic brain damage such as epilepsy or the presence of random brain discharges.<br>8. Those who have undergone electrical or magnetic stimulation within the past 3 months.	1. People suffering from chronic physical diseases.<br>2. Those who have been taking painkillers, tranquilizers, sleep medications, cortisol drugs, anti-epileptic drugs for a long time, treatment of high blood pressure and heart disease drugs, etc.<br>3. Pregnant and breastfeeding women who are currently taking birth control pills.<br>4. Those with a positive family history of mental disorders within two lines and three generations.

Fig. S1. Subject inclusion and exclusion criteria.

B. Gaussian mixture model

The prior distributions of Φ and v are chosen as (S1). The prior distribution of Φ is represented by a Gaussian distribution ($\mathcal{N}$). And the Gamma distribution ($\mathcal{G}$) is applied to denote the prior distribution of v, where a_v and b_v are the hyperparameters of v.

$$p(\Phi) = \prod_{k=1}^{K}\prod_{d=1}^{D}\mathcal{N}(\Phi_{kd}|0, diag(v_k)^{-1}) \qquad (S1a)$$

$$p(v) = \prod_{k=1}^{K}\prod_{p=1}^{P}\mathcal{G}(v_{kp}|a_v, b_v) \qquad (S1b)$$

The prior distributions of γ and ω are Gamma distribution prior as (S2). The two-level probability prior representation is able to obtain a sparse solution and the long-tail property of the student T distribution is insensitive to noise.

$$p(\gamma) = \prod_{k=1}^{K} \mathcal{G}(\gamma_k | a_\gamma, b_\gamma) \tag{S2a}$$

$$p(\omega) = \prod_{k=1}^{K} \prod_{m=1}^{M} \prod_{p=1}^{P} \mathcal{G}(\omega_{kmp} | a_\omega, b_\omega) \tag{S2b}$$

The Dirichlet distribution (Dir) is applied to represent the prior distribution of the initial state $\pi = \{\pi_k | \sum_{k=1}^{K} \pi_k = 1, \forall k$ and the transfer matrix $A = \{A_{ki} | \sum_{i=1}^{K} A_{ki} = 1, \forall k, i\}$. The prior distributions of π and A are chosen as (S3), where θ denotes the hyperparameter of the Dirichlet distribution.

$$p(\pi) = \prod_{k=1}^{K} Dir(\pi_k | \theta_{\pi_k}), \theta_{\pi_k} > 0, \forall k \tag{S3a}$$

$$p(A) = \prod_{k=1}^{K} \prod_{i=1}^{K} Dir(A_{ki} | \theta_{A_{ki}}), \theta_{A_{ki}} > 0, \forall k, i \tag{S3b}$$

C. Selection of areas of interest

The dorsolateral superior frontal gyrus, middle frontal gyrus, medial superior frontal gyrus, and 44 additional ROIs outside the PFC (see Table S1) were selected from these AAL regions, which are either related emotional processes or related to auditory tasks.

Table S1. Information on areas of interest

Full name of ROI	Abbreviations	Coordinates			
		Left hemisphere (.L)		Right hemisphere (.R)	
		AAL Number	X × Y × Z	AAL Number	X × Y × Z
Superior frontal gyrus, dorsolateral	SFGdor	3	-19.7×34.5×41.8	4	20.1×31.6×44.0
Middle frontal gyrus	MFG	7	-34.5×33.0×35.2	8	36.3×33.0×33.7
Superior frontal gyrus, medial	SFGmed	23	-5.7×49.3×30.7	24	8.3×50.7×30.0
Superior frontal gyrus, orbital part	ORBsup	5	-17.5×47.1×-13.5	6	17.2×47.8×-14.2
Middle frontal gyrus, orbital part	ORBmid	9	-31.5×50.0×-9.8	10	31.9×52.2×-10.6
Inferior frontal gyrus, opercular part	IFGoperc	11	-49.2×12.4×18.9	12	48.9×14.6×21.2
Inferior frontal gyrus, triangular part	IFGtriang	13	-46.3×30.1×13.8	14	49.6×30.1×14.5
Inferior frontal gyrus, orbital part	ORBinf	15	-36.7×30.8×-12.0	16	40.0×32.3×-12.0
Olfactory cortex	OLF	21	-9.4×15.3×-12.0	22	8.3×16.1×-11.3
Superior frontal gyrus, medial orbital	ORBsupmed	25	-6.4×54.4×-7.6	26	7.6×51.5×-6.9

Insula	INS	29	-35.9×6.5×3.5	30	37.8×6.5×2.0
Anterior cingulate and paracingulate gyri	ACG	31	-5.0×35.3×13.8	32	7.6×36.7×16.0
Median cingulate and paracingulate gyri	DCG	33	-6.4×-14.9×41.8	34	6.8×-9.0×39.6
Posterior cingulate gyrus	PCG	35	-5.7×-42.9×24.8	36	6.8×-42.2×21.9
Parahippocampal gyrus	PHG	39	-21.9×-15.6×-20.9	40	24.5×-15.6×-20.9
Superior occipital gyrus	SOG	49	-17.5×-84.2×28.5	50	23.1×-80.5×30.7
Middle occipital gyrus	MOG	51	-33.0×-80.5×16.0	52	36.3×-79.8×18.9
Inferior occipital gyrus	IOG	53	-37.4×-78.3×-7.6	54	37.1×-82.0×-7.6
Superior parietal gyrus	SPG	59	-24.1×-59.9×58.8	60	25.3×-59.1×62.5
Angular gyrus	ANG	65	-44.8×-60.6×35.9	66	44.4×-59.9×38.9
Precuneus	PCUN	67	-7.9×-56.2×47.7	68	9.0×-56.2×44.0
Heschl gyrus	HES	79	-43.3×-18.6×10.1	80	44.4×-17.8×10.8
Superior temporal gyrus	STG	81	-54.4×-20.8×7.1	82	57.0×-21.5×7.1
Middle temporal gyrus	MTG	85	-56.6×-34.1×-2.4	86	56.2×-37.0×-1.7
Inferior temporal gyrus	ITG	89	-50.7×-28.2×-23.1	90	52.6×-31.1×-22.4
